# Profiling microRNA from Brain by Microarray in a Transgenic Mouse Model of Alzheimer's Disease

**DOI:** 10.1155/2017/8030369

**Published:** 2017-09-19

**Authors:** Lin-lin Wang, Li Min, Qing-dong Guo, Jun-xia Zhang, Hai-lun Jiang, Shuai Shao, Jian-guo Xing, Lin-lin Yin, Jiang-hong Liu, Rui Liu, Shui-long Guo

**Affiliations:** ^1^Institute of Materia Medica, Chinese Academy of Medical Sciences & Peking Union Medical College, Beijing 100050, China; ^2^Department of Gastroenterology, Beijing Friendship Hospital, Capital Medical University, National Clinical Research Center for Digestive Disease, Beijing Key Laboratory for Precancerous Lesion of Digestive Disease, Beijing 100050, China; ^3^Key Laboratory of Uighur Medicine of Xinjiang Uygur Autonomous Region, Xinjiang Institute of Materia Medica, Urumqi 830004, China; ^4^Department of Neurology, Beijing Tiantan Hospital, Capital Medical University, Beijing 100050, China; ^5^Department of Neurology, Xuanwu Hospital, Capital Medical University, Beijing 100053, China

## Abstract

MicroRNAs (miRNAs) are small noncoding RNAs, which regulate numerous cell functions by targeting mRNA for cleavage or translational repression, and have been found to play an important role in Alzheimer's disease (AD). Our study aimed to identify differentially expressed miRNAs in AD brain as a reference of potential therapeutic miRNAs or biomarkers for this disease. We used amyloid precursor protein (APP) and presenilin 1 (PS1) double transgenic mice and age-matched wild-type (WT) littermates to determine the expression of miRNAs in the brain. MiRNAs were profiled by microarray, and differentially expressed miRNAs underwent target prediction and enrichment analysis. Microarray analysis revealed 56 differentially expressed miRNAs in AD mouse brain, which involved 39 miRNAs that were significantly upregulated and 19 that were downregulated at different ages. Among those miRNAs, a total of 11 miRNAs, including miR-342-3p, miR-342-5p, miR-376c-3p, and miR-301b-3p, were not only conserved in human but also predicted to have targets and signaling pathways closely related to the pathology of AD. In conclusion, in this study, differentially expressed miRNAs were identified in AD brain and proposed as biomarkers, which may have the potential to indicate AD progression. Despite being preliminary, these results may aid in investigating pathological hallmarks and identify effective therapeutic targets.

## 1. Introduction

Alzheimer's disease (AD), the main cause of dementia, is a progressive neurodegenerative disease that is characterized by extracellular senile plagues, intracellular neurofibrillary tangles (NFTs), and neuron loss [1, 2]. Accumulation of the amyloid beta peptide (A*β*) within the brain, which is cleaved by *β*-secretase (BACE1) and *γ*-secretase from amyloid precursor protein (APP), is considered a major cause of AD [3]. Aggregation of A*β* can disturb neurotransmission and cause synaptic impairment [4]. A*β* oligomers trigger the immune system, leading to immune responses, such as the release of chemokines, proinflammatory cytokines, and complement factors. These will increase neuronal death and neuronal synapse loss [5–7]; therefore, the burden of A*β* in AD brain could be an indication of cognitive decline. Several factors have been confirmed to be connected with AD, such as mitochondrial damage, synaptic loss, A*β* accumulation, tau phosphorylation, neuroinflammation, and gene mutations [8–12]. Currently, no effective drugs or treatments exist that can prevent the progress of the disease, and most candidate drugs that targeted A*β* and tau failed during clinical or preclinical research. In addition, there are no noninvasive biomarkers of AD. Thus, microRNAs (miRNAs), which can be easily detected and are widely distributed, have become interesting in AD biomarker research.

MiRNAs are small single-stranded noncoding RNAs, consisting of about 20 nucleotides in length that are widely distributed in cells, blood, serum, and plasma, and regulate a myriad of cell functions [13–15]. Primary miRNA in the nucleus is processed into precursor-miRNA by the RNase III enzyme Drosha and transported to the cytoplasm where it is further processed by an enzyme Dicer to become mature miRNA [12, 16]. Binding to the 3′ untranslated region (3′-UTR) of target mRNA will result in the formation of a silencing complex, in which miRNA regulates the translation process and inhibits the protein from being generated [17]. About 70% of all reported miRNAs can be found in the brains, and several of these miRNAs have been proven to be tightly connected with AD through binding to the *β*-site amyloid precursor protein cleaving enzyme BACE1 or other mRNAs in the amyloidogenic pathways to decrease overproduction of A*β* [18, 19].

MiRNAs are reported to regulate cell functions, including A*β* processing, tau translating, apoptosis, inflammation, and cell cycle phase. Aberrant expression of miRNAs can affect the development and progression of AD [1, 19, 20]. miR-29a, miR-29c, and miR-124 have been shown to interact with the 3′-UTR of BACE1 mRNA, and downregulation of miR-29a, miR-29c, and miR-124 in AD leads to overproduction of A*β* [21–23]. MiR-34a has also been reported to directly bind to tau mRNA and thereby changes the expression of miR-34a in AD, which is important in the expression of tau protein [24].

MiRNA microassay provides an efficient tool for high-throughput and quantitative detection with excellent reproducibility. We detected 3100 capture probes, covering all human, mouse, and rat miRNAs annotated in miRBase 18.0, as well as all viral miRNAs related to these species in APP and presenilin 1 (PS1) double transgenic mouse brain compared with age-matched wild-type (WT) controls, and analyzed any changes to identify aberrant expression of miRNAs.

## 2. Materials and Methods

### 2.1. Animal and Tissue Preparation

Heterozygous APPswe/PS1Δ9 transgenic founder mice were purchased from Jackson Laboratory (Bar Harbor, ME, USA). Age-matched WT littermates were used as controls. All animals received care according to the* Guide for the Care and Use of Laboratory Animals*. Mice were maintained at a constant ambient temperature and a 12 h light/dark cycle, with free access to food and water. Mice were divided into the following groups: 1-month-old, 3-month-old, 6-month-old, and 9-month-old APP/PS1 mice (abbreviated to AD mice) and age-matched WT control mice. Each group contained 6 mice (three males and three females per group). From 3 mice, brains were collected and immediately frozen into liquid nitrogen. The remaining mice were used for additional gene and protein measurements. Both the initial division of mice into different groups and the subsequent selection of mice for gene and protein experiments were done randomly.

### 2.2. RNA Isolation

Total RNA was isolated using TRIzol (Invitrogen, Carlsbad, CA, USA) and purified using an RNeasy Mini Kit (QIAGEN, Dusseldorf, Germany) according to the manufacturer's instructions. RNA quality and quantity were measured using a Nanodrop spectrophotometer (ND-1000, Nanodrop Technologies, Wilmington, Delaware) and RNA integrity was determined by gel electrophoresis.

### 2.3. miRNA Labeling and Array Hybridization

RNA labeling and array hybridization were carried out according to Exiqon's manual (Exiqon, Vedbæk, Denmark). After preparation of the quality control, the miRCURY™ Hy3™/Hy5™ Power labeling kit (Exiqon, Vedbæk, Denmark) was used for miRNA labeling according to the manufacturer's guidelines. A total of 1.0 *μ*L total RNA and 2.0 *μ*L water were combined with 1.0 *μ*L calf-intestinal alkaline phosphatase (CIP) (Exiqon, Vedbæk, Denmark) with the CIP buffer and incubated for 30 min at 37°C, followed by incubation for 5 min at 95°C. Then, a mixture, containing 3.0 *μ*L labeling buffer, 1.5 *μ*L fluorescent labels (Hy3), 2.0 *μ*L DMSO, and 2.0 *μ*L labeling enzyme, was added and incubated for 1 h at 16°C, followed by incubation for 15 min at 65°C to terminate the labeling. After stopping the labeling procedure, the Hy3-labeled samples were hybridized onto the miRCURY LNA array (v.18.0) (Exiqon, Vedbæk, Denmark) according to array manual. The Hy3-labeled samples and 25 *μ*L hybridization buffer were denatured for 2 min at 95°C and hybridized to the microarray for 16 h–20 h at 56°C in a 12-Bay Hybridization System (Hybridization System, Nimblegen Systems, Inc., Madison, WI, USA) following several times of washing using a wash buffer kit (Exiqon, Vedbæk, Denmark). Next, the slides were scanned by the Axon GenePix 4000B microarray scanner (Axon Instruments, Foster City, CA, USA).

### 2.4. Data Analysis

Scanned images were imported into GenePix Pro 6.0 software (Axon Instruments, Union City, CA, USA) for grid alignment and data analysis. Replicated miRNAs were averaged and miRNAs with an intensity ≥ 30 in all samples were chosen for calculating the normalization factor using median normalization. Significantly differentially expressed miRNAs between two groups were identified by fold change and *P* value, and fold change ≥ 2.0 and *P* value ≤ 0.05 were considered significant. Differentially expressed miRNAs between two samples were filtered through fold change. Finally, hierarchical clustering was performed to identify distinguishable miRNA expression profiling among samples.

### 2.5. miRNA Target Prediction

For miRNA target prediction, the target prediction programs, involving TargetScan, miRDB, and miRanda, were used. The search was performed on the 3′-UTR regions of target mRNAs with a *P* value of 0.05 defining the probability distribution of random matches set in the software with a minimum miRNA seed length of 7 [25, 26].

### 2.6. Functional Classification and Pathway Analysis

To further investigate the roles of miRNAs in the process of AD, gene ontology (GO) and Kyoto Encyclopedia of Genes and Genomes (KEGG) pathway analysis were carried out using DAVID (https://david.ncifcrf.gov/). Targets were synthesized and intersections of miRDB, TargetScan, and miRanda databases of the 11 most significant changed miRNAs were taken, and then GO and KEGG analysis were performed.

## 3. Results

### 3.1. Significant Expression Changes of miRNAs in APP/PS1 Mice

**Figure 1:**
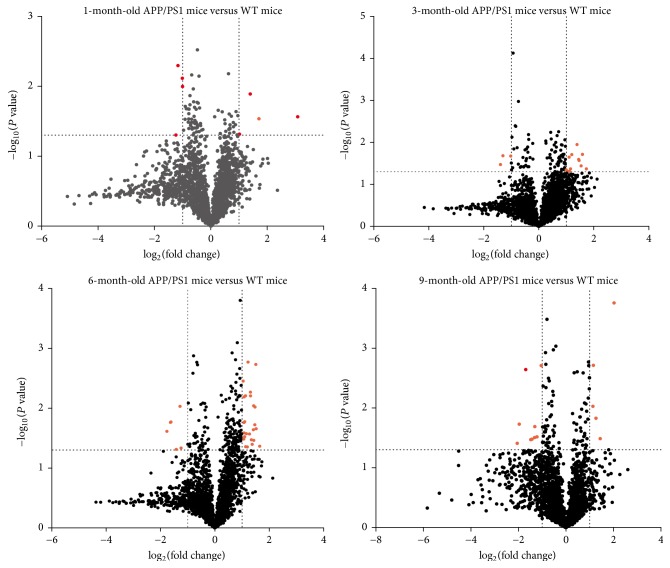
Scatter plot comparison of the fold changes and *P* values of microarray experiments. The vertical lines correspond to 2-fold up and down, respectively, and the horizontal line represents a *P* value of 0.05. The red point in the plot represents the differentially expressed miRNAs with statistical significance.

**Figure 2:**
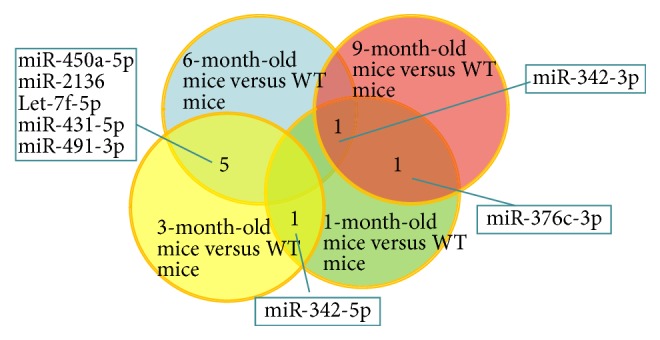
Venn diagram showing the number of miRNAs significantly changed and intersection of changed miRNAs between each age stage. Green areas show the number of changed miRNAs of 1-month-old mice. Yellow areas demonstrate the number of differentially expressed miRNAs in 3-month-old mice. Blue areas display the number of changed miRNAs in 6-month-old mice. Red areas show the number of differentially expressed miRNAs in 9-month-old AD mice. The intersections indicate the miRNAs that changed in two groups or miRNAs that varied in three groups.

To determine the miRNA levels in the brain, we evaluated the miRNAs in 1-month-old, 3-month-old, 6-month-old, and 9-month-old APP/PS1 mice and age-matched WT controls. APP/PS1 mice were analyzed by computational approaches and compared with age-matched WT controls (Figure 1). In contrast with the WT control group, 4 miRNAs were significantly upregulated and 4 were downregulated in the 1-month-old APP/PS1 mouse brain group. In addition, 11 miRNAs were upregulated and 3 downregulated in 3-month-old APP/PS1 mouse brain, 24 miRNAs were upregulated and 6 downregulated in 6-month-old APP/PS1 mouse brain, and 5 miRNAs were upregulated and 9 downregulated in 9-month-old APP/PS1 mouse brain (Table 1). Among these differentially expressed miRNAs, miR-342-3p was consistently upregulated in 1-month-old, 6-month-old, and 9-month-old APP/PS1 mice, which suggests that miR-342-3p may play a role in the process of AD. Moreover, miR-342-5p was elevated in both 1-month-old and 3-month-old APP/PS1 mouse groups, suggesting that miR-342-5p may play a role in early stages of AD. Furthermore, levels of miR-2136, let-7f-5p, miR-431-5p, and miR-491-3p were higher in both 3-month-old and 6-month-old APP/PS1 mouse brain, indicating their potential involvement in the progression of AD (Figure 2).

### 3.2. Function and Pathway Analysis

**Figure 3:**
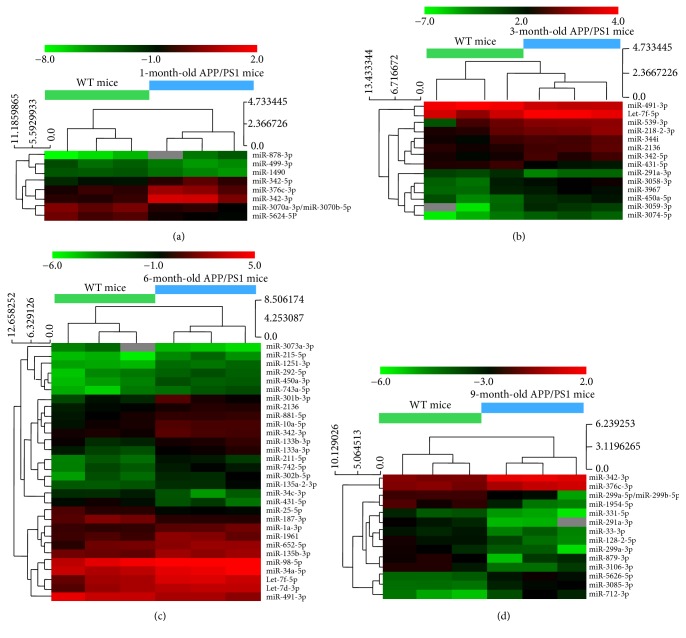
Two-way hierarchical clustering of miRNAs and brain samples. Each row represents a miRNA and each column represents a brain sample. Wide-type control mice are indicated as WT mice. Different age-stage AD mice are shown as 1-month-old (a), 3-month-old (b), 6-month-old (c), and 9-month-old (d) APP/PS1 mice. Clustering tree is shown on the left, and the sample clustering tree appears at the top. The color scale shown at the top illustrates the relative expression level of a miRNA in a certain age group. Red color represents a high relative expression level and a green color represents a low relative expression level.

To explore the potential functions of the significantly different expression of miRNAs, several bioinformatical analyses were performed.  Figure 3 presents the clustering of the miRNAs per age group. For further analysis, we chose 11 evidently different miRNAs that were conserved between both human and mouse: miR-342-3p, miR-342-5p, miR-376c-3p, miR-301b-3p, let-7f-5p, miR-539-3p, miR-491-3p, miR-10a-5p, miR-98-5p, miR-652-5p, and miR-34a-5p.

**Figure 4:**
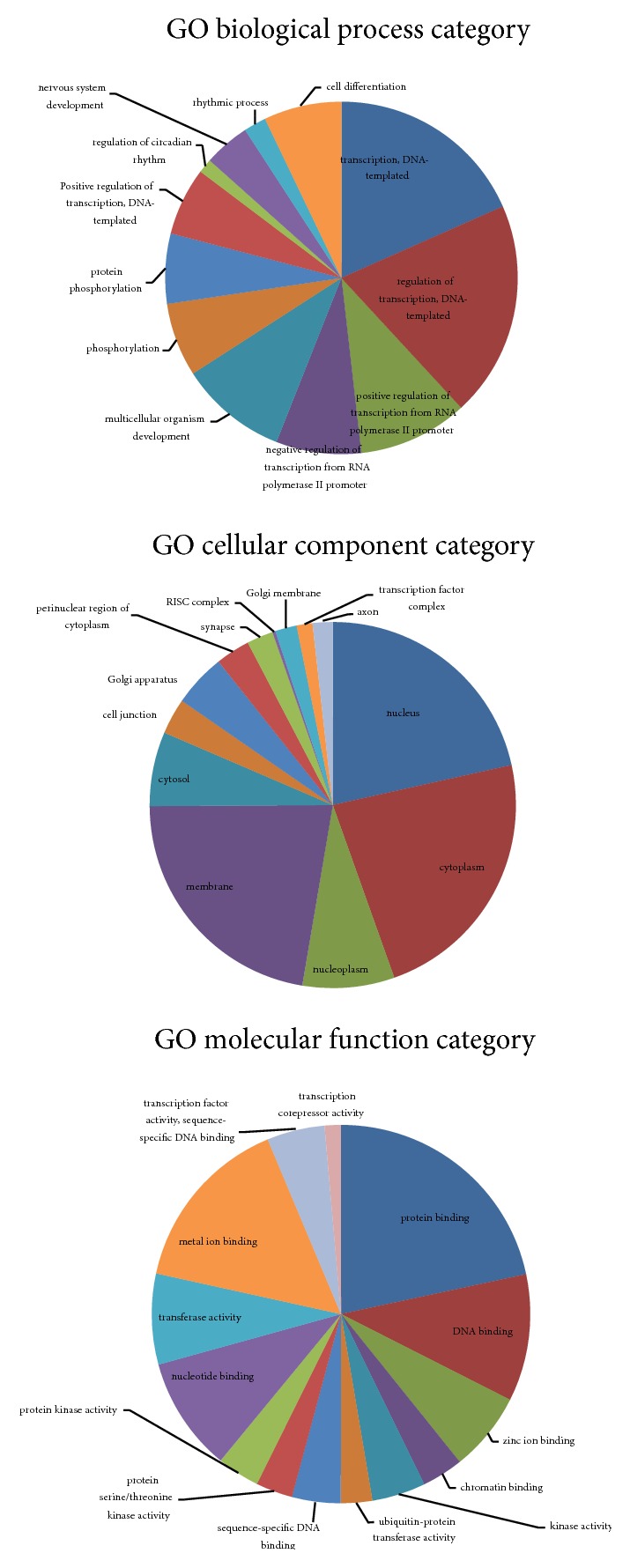
GO enrichment analysis of targets of selected miRNAs carried out with DAVID.

**Figure 5:**
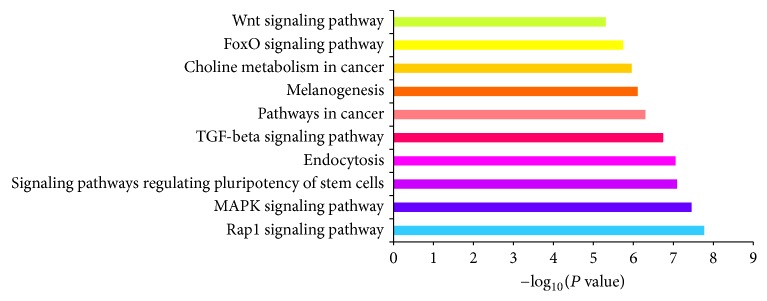
Top 10 significantly enriched pathways of selected 11 miRNAs analyzed with KEGG.

GO and KEGG pathway analyses were performed to elucidate the biological function of the 11 selected miRNAs. GO terms covered 3 domains: molecular function, biological process, and cellular component.  Figure 4 shows the top 12 significantly enriched GO terms at each domain. Moreover, in Figure 5, the 10 most significantly enriched pathways are shown that were mapped with KEGG pathway analysis: the Rap1 signaling pathway, MAPK signaling pathway, signaling pathways regulating pluripotency of stem cells, endocytosis, TGF-beta signaling pathway, pathways in cancer, melanogenesis, choline metabolism in cancer, FoxO signaling pathway, and the Wnt signaling pathway.

### 3.3. Prediction Targets of miRNAs with Different Expressions in AD Brain

To further elucidate the function of miRNAs and the roles of their target genes, we consulted the miRBase (http://www.mirbase.org/) and TargetScan (http://www.targetscan.org/) and summarized the miRNAs and their targets that tightly correspond to AD (Table 2).

Most of the miRNAs were targeted on APP processing and some of those directly combined with 3′-UTR of BACE1 mRNA. The downregulation of miR-1251-5p and miR-34a-5p might alleviate cognitive deficit by increasing the activity of disintegrin and metalloprotease 10 (ADAM10), a major *α*-secretase in brain that is capable of attenuating the production of A*β* by cleavage of APP, resulting in soluble APP*α* [27]. MiR-342-5p, miR-3058-3p, let-7f-5p, miR-1961, miR-301b-3p, miR-98-5p, miR-1251-5p, miR-215-5p, miR-881-5p, miR-135a-2-3p, and miR-33-3p may regulate the expression of insulin-like growth factor 1 (IGF1) or insulin-like growth factor 2 (IGF2), two molecules that could rescue behavior and memory deficits via lowering A*β* levels [28, 29]. The other miRNAs may participate in the phosphorylation of tau, which was the main cause of NFTs. Tau phosphorylation is mainly regulated by glycogen synthase kinase 3*β* (GSK3*β*) while Type 2A protein phosphatase (PP2A) modulates the dephosphorylation of tau protein [30, 31]. miR-376c-3p and miR-342-3p were predicted to affect tau phosphorylation by directly targeting the 3′-UTR of PP2A. Phosphatase and tensin homolog deleted on chromosome ten (PTEN) has been shown to inactivate the phosphatidylinositol 3-kinase (PI3K)/Akt signaling pathway and the absence of PTEN elevates tau aggregation [32, 33]. Several miRNAs derived from our microarray analysis targeted PTEN, such as miR-376c-3p, miR-342-3p, let-7f-5p, miR-10a-5p, miR-301b-3p, miR-98-5p, miR-1251-5p, and miR-34a-5p.

## 4. Discussion

AD is a common form of dementia, with a fast growing morbidity with the speeding aging of the population all over the word. AD patients suffer not only from cognitive dysfunction, such as impairment of memory, visuospatial, language, and executive functions, but also from other psychological symptoms including depression [34]. Therefore, AD patients will gradually lose the ability to take care of themselves and suffer from both mental and psychological distress [35]. However, there are neither effective therapeutic strategies nor early diagnostic biomarkers for the treatment of AD. In addition, in most patients, AD is hardly recognized in the early stages of the disease, and therefore the therapeutic opportunity is often missed because of the delay in diagnosis. miRNAs that regulate posttranscription of proteins have been reported to specifically change in the process of AD and can be easily detected by microarray analysis or RT-PCR. Therefore, miRNAs could potentially serve as noninvasive biomarkers and therapeutic targets of AD [36].

To confirm the variation in miRNA expression at different stages of AD, we chose 1-month-old, 3-month-old, 6-month-old, and 9-month-old AD mice, which were used for microarray analysis. Several differentially expressed miRNAs were found in different stages of AD. Of these different miRNAs, 28 were conserved between human and mouse brain: miR-376c-3p, miR-342-5p, miR-342-3p, miR-499-3p, miR-450a-5p, let-7f-5p, miR-218-2-3p, miR-539-3p, miR-431-5p, miR-491-3p, miR-10a-5p, miR-301b-3p, miR-302b-5p, let-7d-3p, miR-211-5p, miR-98-5p, miR-1251-5p, miR-133a-3p, miR-652-5p, miR-215-5p, miR-34a-5p, miR-135b-5p, miR-34c-3p, miR-187-3p, miR-25-5p, miR-376c-3p, miR-128-2-5p, and miR-331-5p. Considering a probe signal of over 100 as abundance, eleven of the 28 miRNAs (miR-342-3p, miR-342-5p, miR-376c-3p, miR-301b-3p, let-7f-5p, miR-539-3p, miR-491-3p, miR-10a-5p, miR-98-5p, miR-652-5p, and miR-34a-5p) were shown to have targets that are tightly related to AD and could easily be detected. Among these 11 miRNAs, only miR-491-3p was significantly downregulated, while the others were prominently upregulated.

In these differentially expressed miRNAs, miR-342-3p inhibited cell proliferation, migration, and invasion in osteosarcoma, gliomas, and other cancers [37–39]. miR-491 plays a role in different types of cancers, such as glioblastoma and tongue cancer [40, 41]. However, no reports have been published that linked the two miRNAs to AD. To our knowledge, this is the first study to identify the potential effects of miR-342-3p, miR-491-3p, miR-539-3p, miR-376c-3p, miR-10a-5p, and miR-652-5p in the progression of AD. miR-342-5p was reported to manage neuron stem proliferation and differentiation by targeting Notch signaling [42]. It was shown to be upregulated in AD mouse brain and predicted to hamper the function of axon initial segment by decreasing the expression of ankyrin G. Predicted to target BACE1 and IGF2, miR-342-5p may play a significant role in AD. In our findings, let-7f-5p levels were higher in both 3-month-old and 6-month-old APP/PS1 mouse brain, compared to older mice. Unlike our results, let-7f-5p was not significantly different in plasma from AD patients and healthy controls [43]. Thus, we speculate that let-7f-5p may have a different tissue distribution and developmental expression in brain and plasma. miR-301b promoted cell migration and invasion in bladder cancer through downregulating the expression of PTEN, which suggests that miR-301b-3p might target the 3′-UTR of PTEN and regulate the aggregation of tau [44]. miR-98-5p has been proven to regulate the expression of A*β* and might be a novel target of AD [45]. Consistent with the results of a previous study, which illustrated the alteration in plasma samples from AD patients [46], miR-34a-5p showed remarkable upregulation in 6-month-old AD mouse brain and was predicted to be positively connected to the APP process. Therefore, miR-34a-5p could be a potential biomarker for AD. Among the targets of the differentially expressed miRNAs (Table 2), IGF1 and IGF2 were predicted to be the proteins that are affected by a large scale of those differentially expressed miRNAs, such as miR-1961, let-7f-5p, and miR-135a-2-3p. IGF1 and IGF2 are receptors of insulin, and insulin plays an important role in AD by activating the PI3K/Akt signaling pathway and Ras/mitogen activated kinase (MAPK) cascades [47]. Taken together, miRNAs alleviate AD impairment by modulating the expression of IGF1 and IGF2, and therefore the insulin pathway may be a potential target of AD.

To deepen our understanding of these differentially expressed miRNAs, we performed two types of enrichment analyses: GO and KEGG. In this study, the analyses are focused upon the differential expression characteristics of those miRNAs involved in the pathological pathways of AD, to better understand the underlying mechanisms of miRNA and predicted target transductions in this disease. These bioinformatics analyses enabled us to identify many putative signaling pathways in AD pathology in which the differentially expressed miRNAs are involved. These pathological pathways cover the nerve system development process, synapse component, and MAPK signaling pathways. Long-term potentiation (LTP) with 16 counts of total predicted targets and axon guidance with 22 counts also showed significant enrichment, which is not reflected in Figure 4. Combined with the target prediction analysis, we suggest that the 11 selected miRNAs may play a role in AD by regulating the activity in neuronal molecular events that are related to amyloidogenic processes, tau aggregation, and neuron injury.

Other than performing experiments on blood and cerebrospinal fluid (CSF), we specifically explored the variation of miRNAs in brain, which directly reflected the changes of miRNA in AD brain. Moreover, we explored four age groups, including early stage, medium stage, and late stage of AD, and analyzed the variation of miRNA expression in these four groups. Although it is challenging to compare our results to the results of other studies in which different tissues and species were used, several miRNAs, including let-7f-5p, miR-342-5p, and miR-98-5p, showed a similar trend in human blood and CSF [48, 49]. In addition, some differences were found between those results; for example, the level of miR-342-3p was downregulated in AD patient plasma samples, while in our study it was significantly elevated in APP/PS1 mouse brain of 1-month-old, 6-month-old, and 9-month-old mice [46].

miRNA microassay achieved high-throughput screening of miRNAs, and 11 significantly changed miRNAs conserved between human and mouse brain were found in AD brain. Moreover, the alterations of these miRNAs indicate a different development process of AD as indicated by miRNA expression levels at different ages. Among these miRNAs, miR-342-3p was upregulated from 1-month-old to 9-month-old AD mice, suggesting that it may play a critical role in the entire AD process. miR-342-5p was upregulated in brain tissue of 1-month-old and 6-month-old mice, indicating that it is effective at early onset and therefore may act as a specific early biomarker of AD. miR-2136, let-7f-5p, miR-431-5p, and miR-491-3p were upregulated in both 3-month-old and 6-month-old AD mice, implying their role in the development of AD. Combined with subsequent target prediction and enrichment analysis, the 11 miRNAs were predicted to be closely related to the molecular events of the amyloidogenic process, tau phosphorylation, and MAPK and TGF-*β* signaling pathways. Although these miRNAs need to be confirmed due to clearing of false-positive reactions, these findings illustrate that intervention of these miRNAs may affect the signaling transductions from transcriptional to posttranscriptional events and play a role in different processes of AD. In the near future, clinical samples, such as plasma and CSF, are required to detect miRNA levels, targets, and signaling pathways predicted in our study. Thus, our study is a link between the microarray results translating from an animal model to patients who are diagnosed with AD and have developed cognitive deficits with pathological alterations of extracellular A*β* deposition and intracellular NFTs.

## 5. Conclusions

In summary, this study was also the first miRNA microarray using different-stage AD brain tissue, and through bioinformatics analysis, eleven miRNAs were shown to have plentiful altered expressions and to be conserved in evolution with possible target correlation to AD and suggested to act as “markers” to distinguish the pathological changes of this disease. Our findings provided a clue for understanding the pathological hallmarks and looking for effective therapeutic targets in the miRNA-mRNA level of AD.

## Figures and Tables

**Table 1:** Changes in expression of miRNAs in 1-month-old, 3-month-old, 6-month-old, and 9-month-old APP/PS1 mice as compared to age-matched WT controls.

	Change	miRNA ID	miRNA name	Fold change	*P* value
1-month-old APP/PS1 mice versus WT control	Up	30681	mmu-miR-376c-3p	2.023	0.048
		42576	mmu-miR-342-5p	2.636	0.012
		42646	mmu-miR-878-3p	8.470	0.027
		32884	mmu-miR-342-3p	3.266	0.029
	Down	146015	mmu-miR-1940	0.446	0.005
		168826	mmu-miR-5624-5p	0.500	0.010
		145692	mmu-miR-499-3p	0.420	0.047
		148259	mmu-miR-3070a-5p/mmu-miR-3070b-5p	0.497	0.008

3-month-old APP/PS1 mice versus WT control	Up	148453	mmu-miR-3074-5p	2.731	0.03
		17835	mmu-miR-450a-5p	2.621	0.011
		146004	mmu-miR-2136	2.146	0.023
		148047	mmu-miR-3058-3p	2.761	0.027
		42576	mmu-miR-342-5p	2.066	0.049
		169158	mmu-miR-344i	2.163	0.049
		17752	mmu-let-7f-5p	2.297	0.020
		46728	mmu-miR-3059-3p	3.315	0.043
		145836	mmu-miR-218-2-3p	2.910	0.036
		148074	mmu-miR-539-3p	3.002	0.019
		169301	mmu-miR-3967	2.224	0.043
	Down	145705	mmu-miR-431-5p	0.405	0.021
		42595	mmu-miR-291a-3p	0.493	0.021
		147701	mmu-miR-491-3p	0.381	0.034

6-month-old APP/PS1 mice versus WT control	Up	146199	mmu-miR-1961	2.696	0.034
		13485	mmu-miR-10a-5p	2.841	0.002
		17835	mmu-miR-450a-5p	2.483	0.006
		42712	mmu-miR-742-5p	2.692	0.009
		146004	mmu-miR-2136	2.057	0.004
		42664	mmu-miR-301b-3p	2.668	0.023
		14189	mmu-miR-302b-5p	3.141	0.043
		145633	mmu-let-7d-3p	2.088	0.033
		19601	mmu-miR-211-5p	2.769	0.019
		11182	mmu-miR-98-5p	2.099	0.017
		32884	mmu-miR-342-3p	2.476	0.005
		27571	mmu-miR-292-5p	2.104	0.026
		46275	mmu-miR-1251-5p	2.329	0.002
		148017	mmu-miR-743a-5p	2.538	0.034
		10916	mmu-miR-1a-3p	2.140	0.017
		146137	mmu-miR-133a-3p	2.868	0.022
		148423	mmu-miR-652-5p	2.230	0.027
		17752	mmu-let-7f-5p	2.782	0.010
		11210	mmu-miR-215-5p	2.589	0.040
		146160	mmu-miR-133b-3p	2.285	0.044
		168586	mmu-miR-34a-5p	2.416	0.027
		42587	mmu-miR-881-5p	2.104	0.006
		46859	mmu-miR-135a-2-3p	2.192	0.044
		145914	mmu-miR-135b-5p	2.186	0.006
	Down	42767	mmu-miR-34c-3p	0.412	0.009
		145705	mmu-miR-431-5p	0.326	0.017
		145637	mmu-miR-187-3p	0.294	0.024
		42929	mmu-miR-25-5p	0.374	0.049
		148276	mmu-miR-3073a-3p	0.322	0.017
		147701	mmu-miR-491-3p	0.422	0.046

9-month-old APP/PS1 mice versus WT control	Up	30681	mmu-miR-376c-3p	2.407	0.015
		42494	mmu-miR-712-3p	2.721	0.033
		32884	mmu-miR-342-3p	4.074	0.0001
		169373	mmu-miR-5626-5p	2.195	0.009
		46297	mmu-miR-3085-3p	2.229	0.002
	Down	148605	mmu-miR-128-2-5p	0.430	0.030
		42595	mmu-miR-291a-3p	0.256	0.019
		148105	mmu-miR-3106-3p	0.359	0.034
		17866	mmu-miR-331-5p	0.403	0.020
		42625	mmu-miR-299a-3p	0.371	0.033
		148525	mmu-miR-1964-5p	0.241	0.039
		145757	mmu-miR-33-3p	0.487	0.002
		42621	mmu-miR-879-3p	0.401	0.031
		11038	mmu-miR-299a-5p/mmu-miR-299b-5p	0.310	0.002

**Table 2:** Significantly changed miRNAs and their predicted targets.

miRNA ID	miRNA name	Target
42576	mmu-miR-342-5p	IGF2, BACE1
148047	mmu-miR-3058-3p	IGF2
17752	mmu-let-7f-5p	IGF1, PTEN
148074	mmu-miR-539-3p	GSK3*β*
42595	mmu-miR-291a-3p	APP
147701	mmu-miR-491-3p	BACE1, BDNF
146199	mmu-miR-1961	IGF1, IGF2
13485	mmu-miR-10a-5p	BDNF, PTEN
42664	mmu-miR-301b-3p	IGF1, LDLR, NAV3, PTEN
19601	mmu-miR-211-5p	BDNF
11182	mmu-miR-98-5p	BACE1, IGF2, PTEN
46275	mmu-miR-1251-5p	IGF1, APBB2, PTEN, ADAM10
148423	mmu-miR-652-5p	BACE1
11210	mmu-miR-215-5p	IGF1
168586	mmu-miR-34a-5p	PTEN, ADAM10
42587	mmu-miR-881-5p	IGF1, APP, IGF2,
46859	mmu-miR-135a-2-3p	IGF2
145757	mmu-miR-33-3p	IGF1
30681	mmu-miR-376c-3p	PTEN, PP2A
32884	mmu-miR-342-3p	PTEN, PP2A
